# A Prospective Multicentre Study to Improve Postoperative Pain: Identification of Potentialities and Problems

**DOI:** 10.1371/journal.pone.0143508

**Published:** 2015-11-24

**Authors:** Esther Pogatzki-Zahn, Patrick Kutschar, Nadja Nestler, Juergen Osterbrink

**Affiliations:** 1 Department of Anaesthesiology, Intensive Care and Pain Medicine, University Hospital Muenster, Muenster, Germany; 2 Institute of Nursing Science and Practice, Paracelsus Medical University, Salzburg, Austria; Azienda Ospedaliero-Universitaria Careggi, ITALY

## Abstract

Many studies still indicate insufficient pain management after surgery, e.g., in patients after small- or medium-size operations. Yet it is still uncertain if postoperative pain based on patient-related outcomes can be improved by implementing guideline-related programmes in a multicentre approach. Adult patients in six hospitals in one German city were included in this prospective study. Data collection took place twice in each hospital, once before and once after implementation of concepts and in-house training. Pain and pain-related aspects were assessed one day after surgery and compared between the pre- and post-test group including subgroup analysis of certain surgical procedures by using Student’s t-tests, Mann-Whitney U tests and chi-square tests (alpha_two-tailed_ = 0.05). Overall, pain at rest and during movement was slightly lower after the intervention. Significant changes were observed after thoracic surgery, small joint surgery and other minor surgical procedures. The rather moderate decrease in pain likely relates to a reasonable pre-existing pain management and to detached improvements in certain patient subgroups. Interestingly, specific analyses revealed significantly lower post-test pain as compared to pre-test pain only in patients without pre-existing chronic pain. Side effects related to pain medication were significantly lower after intervention. Our data show, for the first time, benefits of a perioperative teaching programme in a multicentre approach. Pain ratings improved mainly in specific subgroups of patients, e.g., small surgical procedures and patients without preoperative pain. Thus, general improvement is possible but special attention should be paid to the group of patients with preoperative pain.

## Introduction, Aim and Purpose

Although established guidelines [1, 2] and evidence-based recommendations [3] exist, postoperative pain remains predominantly undertreated [4]. Various studies demonstrate deficits in pain management of up to 50% of postoperative patients regardless of hospital type, site of surgery or country [5–9]. Known barriers are deficient knowledge about pain management, lack of written instructions and insufficient pain assessment among others [10–12].

One of the factors moderating postoperative pain intensity is the extent of the surgical procedure itself. As indicated recently [13], pain in patients after major surgical procedures seems to be lower than pain after minor or medium-sized procedures. One explanation may be the existence of excellent analgesic strategies for major procedures including epidural and regional analgesic techniques. Introduction of such techniques together with the implementation of acute pain services in clinical practice during the last 20 years led to improvements in the treatment of postoperative pain in specific subpopulations, e.g. after major surgical procedures [14]. However, the great majority of postoperative patients do not receive any type of regional anaesthetic techniques, because the surgical procedure was minor and pain expected to be lower. In reality, this assumption is flawed [8, 13], since the occurrence of pain does not depend solely on how minor a surgical procedure may be. The future focus should therefore be not only on the improvement of pain after major procedures but also after minor and medium-sized procedures.

There are ongoing efforts to improve postoperative pain under “real-life” conditions. Besides humanitarian reasons, it is well established that severe postoperative pain is associated with physical problems like pulmonary or cardiac complications, longer hospital stays and higher mortality. In turn, optimal pain management with, for instance, epidural analgesia is able to significantly reduce mortality [15]. Patient dissatisfaction is strongly associated with insufficient postoperative pain relief [16] and severe postoperative pain frequently leads to chronic pain after surgery [17, 18]. Thus, optimal perioperative pain management is a key to increase the physical, mental and social well-being of patients.

Only very few studies address possibilities to improve postoperative pain under real-life conditions. Among those available are either single centre studies [19], studies lacking patient-related outcome data [11] or studies without an intervention [6–8, 13, 20]. In this study, we performed a prospective interventional study with a pre-post-test design to assess improvements of postsurgical pain management based on patient-related outcome in six hospitals in a medium-size German city after implementing guideline related pain concepts and in-house training.

## Methods

### Setting and Design

This prospective study is part of the health care services project ‘Action Alliance Pain-Free City Muenster’, which was conducted from January 2010 to December 2013 with a pre-post-test design. Details of the entire project have been published elsewhere [21]. Postoperative patients in all (six) non-university hospitals of the German city Muenster were included. Each hospital participated twice, once before the intervention (pre-intervention group) and once after the intervention (post-intervention group). All primary parties involved (patients, nurses, physicians, anaesthesiologists) were examined by standardised questionnaires, which were developed for and adjusted to each sample. Data collections, methods and instruments in the pre- as in the post-test were realized identically. This present article explicitly focuses on the pre-post-analysis of patient data.

### Ethical Clearance and Considerations

Ethical clearance was given by the ethics committee of the Institutional Review Board of the University of Muenster (Ref. 2010-010-f-S). All participants were informed about voluntary participation and the guaranteed right to withdraw from the study at any time without repercussions. Respondents gave written consent and were fully informed about their rights to privacy. Data of participants were stored electronically and pseudonymised with randomly generated codes.

### Participants and Sample

In each hospital, all patients on the first day after an elective surgery were included consecutively within a predefined time of four weeks (convenience sample), provided that they matched the inclusion criteria. The surveys took place from Tuesday to Saturday as elective surgeries are realised on weekdays only. Exclusion criteria were refusal to participate, no written consent, age younger than 18 years, cognitive impairment, psychiatric disorders, fragile general state of health (based on diagnoses from ward physician), insufficient German language capabilities or staying in the intensive or intermediate care unit.

### Data Collection and Measures

Data collection for the pre-test phase was carried out by trained study assistants over a period of 4 to 6 weeks in each hospital (May to August 2010). After a six-month intervention phase, the post-test phase took place from September 2011 to February 2012, again over a period of 4 to 6 weeks in each hospital.

#### Collection of Written Policies

First, the status-quo of standard regulations for pain management of each hospital was analysed by the research team. These written policies and regulations combined with the pre-test survey results were used to identify recommendations and intervention packages for each hospital.

#### Questionnaire for Patients

Patients were examined with netbook-based standardised questionnaires by self-interview or interviewed by specially trained research assistants. The 38-item questionnaire was adapted from a prior study [8]. Several items of the prior test survey were revised, adapted, extended and re-tested in a pilot pre-test with a comparable sample of patients in a different city. Pain intensity, perceived side effects due to drug pain therapy and subjective quality rating of the received pain therapy were assessed by items in the applied questionnaire. To measure the intensity of preoperative as well as postoperative pain at rest and pain during movement, the Numeric Rating Scale (NRS, 0–10) was used. Patients evaluated the severity of side effects (nausea, vomiting, fatigue, dizziness, shaking while walking) on a 4-point scale item battery (0–no side effect, 1–mild side effects, 2–moderate side effects, 3–severe side effects).

#### Biometrical and Medical Data

Biometrical (age, sex, weight, body height, Body Mass Index—BMI) and medical (surgical procedure, surgical procedure code (OPS), code of disease (ICD), documented disease, scheduled analgesics & analgesics on demand (PRN), presence of malignant tumour) data were collected directly from the patients’ medical records and clinical data sheets.

#### Measurement of Results

The outcome variables “pain intensity” and “severity of side effects” were treated as ordinal scale measures. For the rated pain intensity on the 11-point NRS, cutoff values [7, 8] were set to >3 (pain at rest) and >5 (pain during movement).

Because the total sample showed heterogeneity relevant to the outcome on postoperative pain, we performed analyses of primary and secondary outcome variables between the pre- versus post-intervention group in pre-defined procedure surgical subgroups. Twelve comparable subgroups with sufficient sample sizes (n_min_ = 14) were built to test differences in pain outcome variables. These surgical subgroups were classified on the basis of the documented surgical procedures and categorized by their location and extent of the surgery (e.g. small, medium and major joint surgery). Pre-post-comparisons were carried out for selected single surgical procedures based on the OPS-codes. Similar pain-associated procedures were assigned to one surgical group resulting in four procedures (OPS hip joint replacement: 5–820; thyroid surgery: 5–061 to 5–063, 5–067, 5–069; hysterectomy: 5–682 to 5–685; radical and expanded excision of diseased mesh from skin and hypoderm: 5–895).

### Intervention Strategy

After the pre-test, a 6-month intervention phase was realized per hospital. First, selected staff members, physicians, nurses and physiotherapists, were trained by a medical and a nursing specialist in pain management, about 8 hours in each hospital. The content of the training was based on the individual results of the pre-test within the hospital in combination with evidence-based recommendations and national legal guidelines (Table 1).

**Table 1:** Training contents during intervention phase.

Content of training sessions
Presentation of the pre-test results
Possibilities of pain assessment regarding self-reporting and external assessment of pain
Recommendations of pharmacological pain therapy (Pain therapy concepts, including scheduled analgesics on demand and cutoff points)
Regulation of the responsibilities for the pain management for all staff members
Possibilities of non-pharmacological strategies of pain management
Recommendations how to educate patients
Strategies of optimization the pain management for each hospital

All hospitals had to convene a committee for pain management to develop written policies for pain management in which the responsibilities of all staff members were defined. Optimizing pain management was planned in each hospital based on pre-test results and individual difficulties. Regulations for pain assessment and documentation tools for the daily inspection of the pain situation were created or adapted. Furthermore, each hospital got an individual proposal for the improvement of the pharmacological and non-pharmacological pain treatment strategies. For instance, cutoff points for adapting the therapy and a schematic approach on how to adequately treat the pain above the cutoff where introduced.

Dissemination and implementation of these strategies within each hospital was provided by the selected staff members within a time frame of 6 months. Again, this training was performed with all occupational groups (nurses, physicians, anaesthesiologists and physiotherapists). The goal was to reach a high and comparable standard of pain assessment, medical documentation and treatment on every surgical ward in all hospitals. These implementations were supported by internal trainings in the hospitals as well as by the research group.

### Statistical Analysis

All statistical analyses were conducted using SPSS 21.0. Sample characteristics were primarily analysed applying descriptive statistics. Pre-post-comparisons for normally distributed variables were executed using Student’s t-test for independent samples. Differences in ordinal or non-normal distributions were statistically tested using Mann-Whitney-U-Test. In case of dichotomous variables, Pearson chi-square analyses and Cramer’s V (V) were exerted. In general, type one error was set to alpha = 0.05 and two tailed p-values were used to assess statistical significance.

## Results

### Demographics

**Fig 1:**
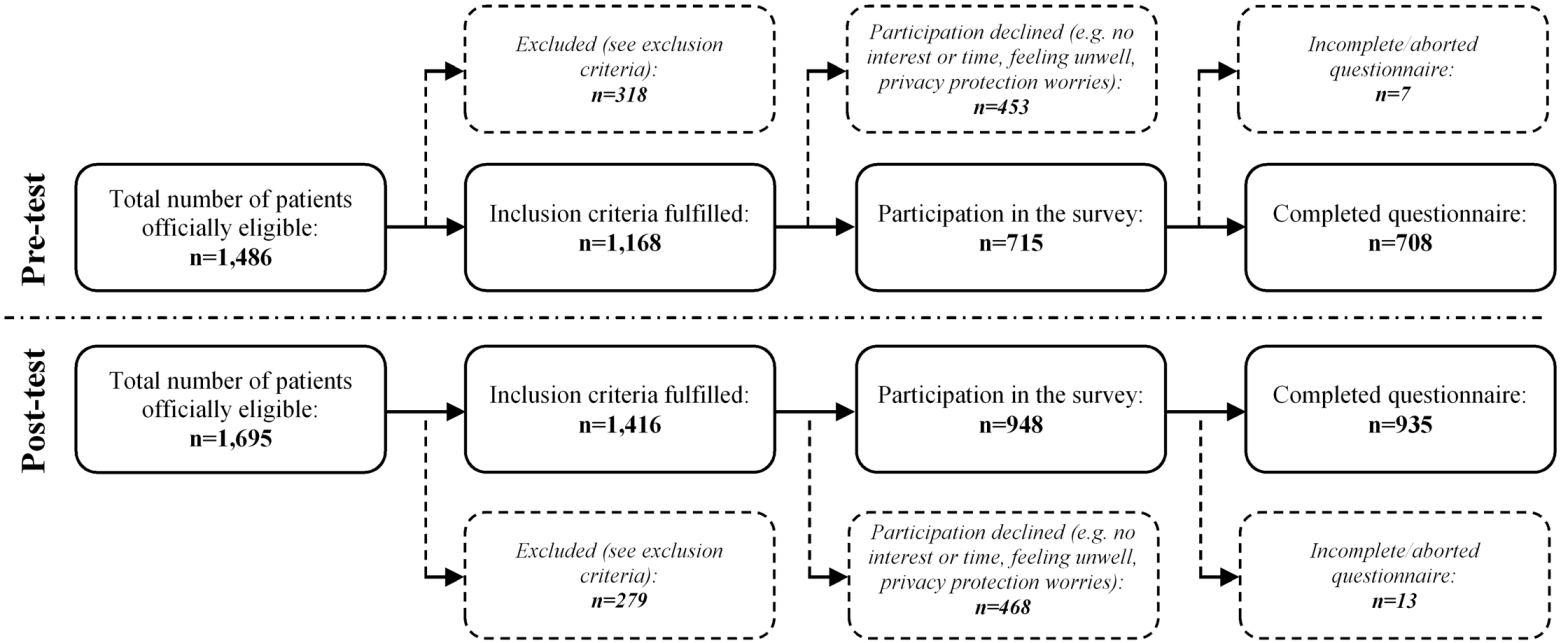
Flow chart of survey response rates for pre- and post-test.

In the pre-intervention period, a total of 1,486 patients from 6 hospitals were screened for participation. As a result of excluding, declined participation and data cleansing (Fig 1), data from 708 patients were eligible for further analysis in the pre-intervention group. In the post-intervention period a total of 935 out of 1,695 screened patients were included for analysis. A comparable percentage of patients fulfilled inclusion criteria in the pre-test period (60.6%) and the post-test group (66.0%). Results of all demographic data of the total sample are summarized in S1 Table.

### Subgroup analysis

Table 2 shows the number of included patients per surgery subgroup. Further detailed characteristics of the patients—separately presented for each surgery group—are given in S2 Table.

**Table 2:** Selected surgery subgroups for pre-post-comparison of pain intensity.

Surgeries	Pre-test (n)	Post-test (n)
Joint (s)	26	64
Joint (m)	28	54
Joint (l)	109	71
Long bone (m, l)	39	21
Spine (m)	28	26
Thorax (s, m, l)	28	43
Vascular (s, m)	15	21
Visceral (m)	82	47
Gynaecology (m)	54	51
Urology (m, l)	42	38
Plastic (skin) (s)	14	40
Tumour (skin) (s)	78	100
**Total (selected surgeries)**	543	576

Notes: (s) small, (m) medium, (l) major surgery

### Pre-Post-Comparison

#### Pain at Rest & Pain during Movement–Comparison in the Total Sample

Pain at rest was significantly higher in the pre-intervention phase (n = 706) compared to the post-intervention phase (n = 917; p≈0.006) (Table 3). Pain during movement was slightly higher in the pre-intervention phase (n = 696) compared to the post-intervention phase (n = 908; p≈0.080). The percentage of patients with pain scores above cutoff-values for pain at rest (NRS>3) were 23.8 in the pre- and 20.8 in the post-intervention group.

**Table 3:** Postoperative pain ratings (NRS) at rest and during movement in the pre-intervention and post-intervention group–total sample and selected surgical groups.

Surgeries	Differences in Pain Intensity—NRS^a^				NRS-cutoff^b^		
	Pre-test mean rank	Post-test mean rank			% > cutoff		
	(x_0.5_, n_valid_)	(x_0.5_, n_valid_)	Z, p	▼ ~ ▲	pre vs. post	V	p (chi²)
**Total Sample**							
Pain at rest	847.6 (2.0, n = 706)	784.6 (2.0, n = 917)	-2.742, **p≈0.006**	▼ **	23.8 vs. 20.8%	0.035	p≈0.153 (2.04)
Pain during movement	825.4 (4.0, n = 696)	784.9 (3.0, n = 908)	-1.750, p≈0.080	▼ ^T^	25.9 vs. 23.0%	0.033	p≈0.188 (1.74)
**Joint (s)**							
Pain at rest	46.6 (1.5, n = 26)	44.3 (1.0, n = 63)	-0.395, p≈0.693	~	34.6 vs. 14.3%	**0.230** *	**p≈0.030** (4.71)
Pain during movement	44.8 (4.0, n = 26)	45.1 (4.0, n = 63)	-0.050, p≈0.960	~	34.6 vs. 25.4%	0.093	p≈0.379 (0.77)
**Joint (m)**							
Pain at rest	39.8 (1.0, n = 28)	42.4 (2.0, n = 54)	-0.467, p≈0.640	~	21.4 vs. 22.2%	0.009	p≈0.934 (0.01)
Pain during movement	37.2 (3.0, n = 28)	43.0 (4.0, n = 53)	-1.064, p≈0.287	~	32.1 vs. 32.1%	0.001	p≈0.995 (0.01)
**Joint (l)**							
Pain at rest	89.4 (2.0, n = 109)	93.4 (3.0, n = 72)	-0.502, p≈0.616	~	30.3 vs. 36.1%	0.061	p≈0.412 (0.67)
Pain during movement	88.4 (4.5, n = 108)	92.4 (5.0, n = 71)	-0.498, p≈0.618	~	37.0 vs. 39.4%	0.024	p≈0.746 (0.11)
**Long bone (m, l)**							
Pain at rest	31.8 (3.0, n = 39)	24.8 (1.0, n = 19)	-1.491, p≈0.136	~	30.8 vs. 21.1%	0.102	p≈0.437 (0.60)
Pain during movement	29.0 (4.0, n = 36)	26.1 (4.0, n = 19)	-0.662, p≈0.508	~	30.6 vs. 15.8%	0.161	p≈0.232 (1.43)
**Spine (m)**							
Pain at rest	29.6 (3.0, n = 28)	25.3 (1.0, n = 26)	-1.022, p≈0.307	~	28.6 vs. 23.1%	0.063	p≈0.645 (0.21)
Pain during movement	27.9 (3.0, n = 27)	26.1 (3.5, n = 26)	-0.422, p≈0.673	~	25.9 vs. 19.2%	0.080	p≈0.560 (0.34)
**Thorax (s, m, l)**							
Pain at rest	42.3 (2.0, n = 28)	30.1 (1.0, n = 41)	-2.538, **p≈0.011**	▼ *	28.6 vs. 12.2%	**0.206** ^T^	**p≈0.088** (2.92)
Pain during movement	36.5 (3.5, n = 28)	34.0 (3.0, n = 41)	-0.518, p≈0.604	~	21.4 vs. 22.0%	0.006	p≈0.959 (0.01)
**Vascular (s, m)**							
Pain at rest	18.2 (0.0, n = 15)	18.7 (0.0, n = 21)	-0.177, p≈0.860	~	6.7 vs. 23.8%	0.227	p≈0.174 (1.85)
Pain during movement	17.5 (2.0, n = 15)	19.2 (2.0, n = 21)	-0.490, p≈0.634	~	6.7 vs. 19.0%	0.176	p≈0.290 (1.12)
**Visceral (m)**							
Pain at rest	64.4 (2.0, n = 81)	64.6 (2.0, n = 47)	-0.025, p≈0.980	~	24.7 vs. 27.7%	0.033	p≈0.711 (0.14)
Pain during movement	65.7 (4.0, n = 82)	63.8 (4.0, n = 47)	-0.288, p≈0.773	~	31.7 vs. 34.0%	0.024	p≈0.785 (0.07)
**Gynaecology (m)**							
Pain at rest	49.3 (2.0, n = 54)	56.9 (3.0, n = 51)	-1.297, p≈0.195	~	25.9 vs. 35.3%	0.102	p≈0.297 (1.09)
Pain during movement	47.1 (4.0, n = 54)	58.4 (5.0, n = 50)	-1.933, **p≈0.053**	▲ ^T^	25.9 vs. 42.0%	**0.170** ^T^	**p≈0.083** (3.00)
**Urology (m, l)**							
Pain at rest	37.6 (0.0, n = 43)	44.9 (2.0, n = 38)	-1.486, p≈0.137	~	11.6 vs. 26.3%	**0.189** ^T^	**p≈0.089** (2.88)
Pain during movement	41.0 (2.5, n = 42)	40.0 (2.0, n = 38)	-0.207, p≈0.836	~	16.7 vs. 15.8%	0.012	p≈0.915 (0.01)
**Plastic (skin) (s)**							
Pain at rest	31.4 (1.0, n = 14)	25.4 (0.0, n = 39)	-1.329, p≈0.184	~	14.3 vs. 10.3%	0.056	p≈0.683 (0.17)
Pain during movement	30.0 (2.0, n = 13)	25.3 (2.0, n = 39)	-0.987, p≈0.323	~	15.4 vs. 5.1%	0.167	p≈0.229 (1.44)
**Tumour (skin) (s)**							
Pain at rest	96.4 (1.0, n = 78)	75.2 (0.0, n = 91)	-2.982, **p≈0.003**	▼ **	19.2 vs. 6.6%	**0.191** *	**p≈0.013** (6.16)
Pain during movement	92.6 (2.0, n = 77)	75.6 (1.0, n = 89)	-2.334, **p≈0.020**	▼ *	13.0 vs. 11.2%	0.027	p≈0.730 (0.12)
**Total (surgeries)**							
Pain at rest	580.1 (2.0, n = 543)	526.8 (1.0, n = 562)	-2.842, **p≈0.004**	▼ **	24.5 vs. 21.0%	0.042	p≈0.165 (1.92)
Pain during movement	560.9 (4.0, n = 536)	533.6 (3.0, n = 557)	-1.443, p≈0.149	~	26.5 vs. 24.6%	0.022	p≈0.472 (0.52)

Notes:

^▼^ significant reduction,

^~^ no difference,

^▲^ significant increase,

*** p<0.001,

** p<0.01,

* p<0.05,

^T^ p<0.10,

(s) small, (m) medium, (l) major,

^a^ Nonparametric Mann-Whitney-U-Test is used to test differences between ordinal distributions,

^b^ Pearson chi-square and Cramer V are used to test differences between dichotomous distributions (pain at rest>3, pain during movement>5)

#### Pain at Rest & Pain during Movement–Comparison in Samples of Surgical Subgroups

In general, unrelated to the pre- or post- intervention group, median pain ratings at rest were 2 or lower and pain ratings during movement were 4 or lower after almost every surgical procedure (Table 3). The most painful procedures were gynaecological and major joint surgeries with median pain ratings during movement up to 5. Accordingly, the percentage of patients with pain above cutoff values was highest in patients after gynaecological (up to 42%) and major joint surgeries (up to 39%). However, the percentage of patients with pain ratings above cutoff values was much less for most other types of surgical procedures.

**Fig 2:**
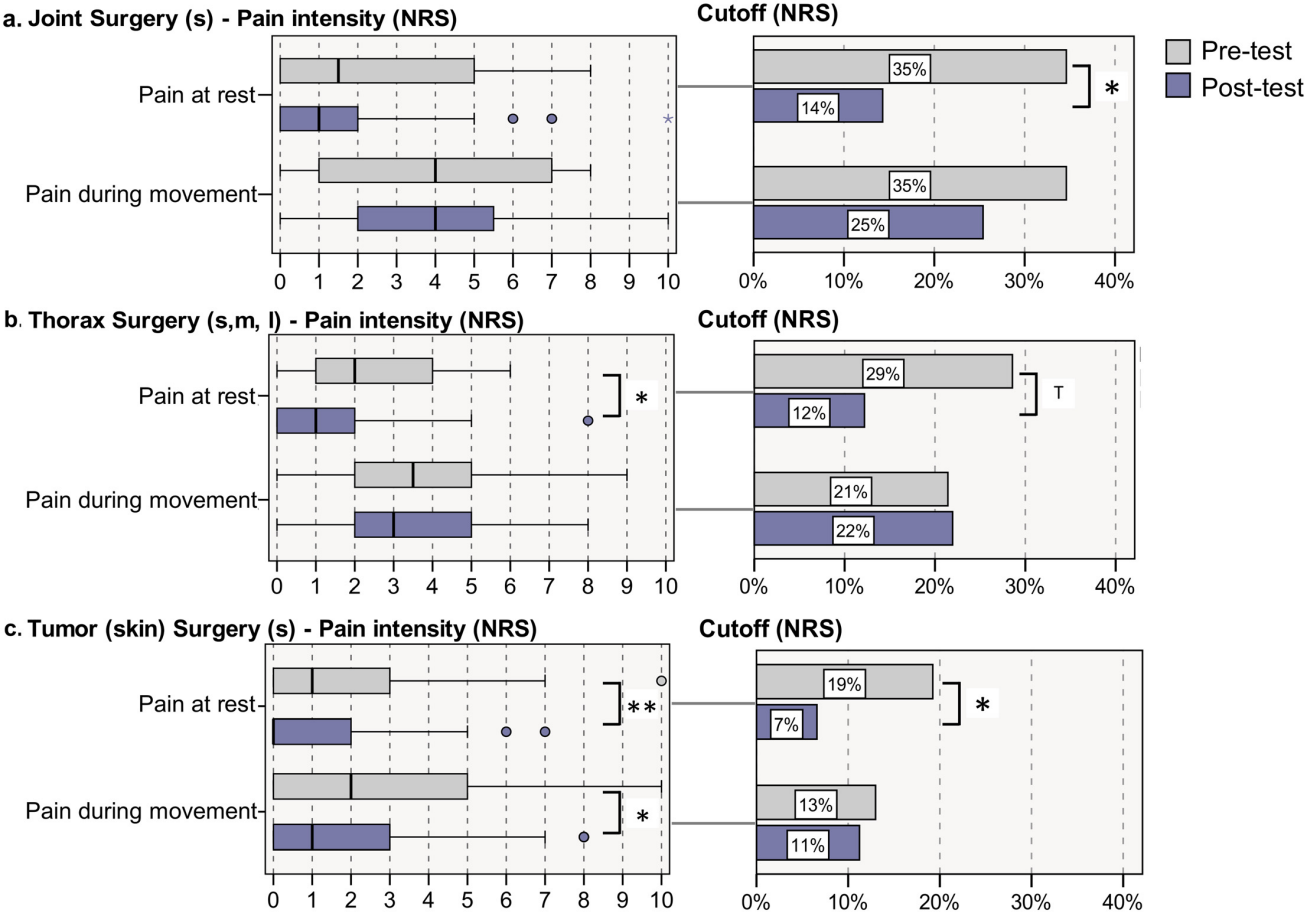
Postoperative pain ratings for those procedures with significant effects due to the intervention after a. joint surgery (s), b. thorax surgery (s, m, l) and c. tumour (skin) surgery (s). Pain intensity distribution is displayed using boxplots and statistically tested with Mann-Whitney-U-test; proportion above NRS-cutoffs is displayed using barcharts and statistically tested with chi-square test; ** p<0.01, * p<0.05, ^T^ p<0.10.

Comparison between pre- and post-intervention groups revealed similar or lower median pain ratings and less patients with pain ratings above cutoff in the post-intervention group compared to the pre-intervention group regardless of the type of surgery (Table 3). Significant different median pain ratings and percentage of patients above cutoff between the post- versus the pre-intervention group are shown in bold in Table 3 and depicted in Fig 2. For instance, patients after small joint surgeries reported significantly less pain at rest (and less patients with pain above cutoff) after the intervention compared to those before intervention (p<0.05) (Table 3 and Fig 2a). A similar pain reduction after intervention was observed for patients after thoracic surgery and after surgeries of the urinary tract (Table 3). And finally, a significant reduction of pain (at rest and during movement) and a reduced percentage of patients above cutoff was evident in patients with minor surgical procedures of the skin (mainly tumour surgery) (Table 3 and Fig 2c). Overall, pain intensities were lower for most of the surgery groups after the intervention. However, a minor trend for more intense pain was observed for medium gynecological surgeries (Table 3).

#### Pain at Rest & Pain during Movement–Comparison in Selected Surgical Procedures

Because reliable pre-post-comparisons are best in groups of patients with exactly the same surgical procedure, we further analysed and compared pre-post-data from single surgical procedures based on the OPS-Codes [13]. As described in the method section, only groups with acceptable number of patients in the pre- and post-intervention groups were considered; four specific surgical procedures were applicable and results are shown in S3 Table. Considerable reductions in pain intensities after intervention were observed for patients with radial and expanded excisions of diseased mesh from skin and hypoderm, in which both pain at rest (p<0.001) and pain during movement (p<0.05) were decreased significantly. Pain at rest and during movement was reduced by trend in thyroidal surgery patients (p<0.10). Descriptive proportions based on clinically relevant NRS-cutoff values reassure these reductions. However, pain intensities of patients with hysterectomies and hip joint replacement remained the same or even increased (p<0.05).

#### Pain at Rest & Pain during Movement–Comparison pre and post Intervention in Patients with and without Preoperative Pain

It has been suggested that patients with chronic pain before surgery are at higher risk to experience severe acute pain after surgery [22]. We therefore compared postoperative pain rating in the pre-intervention and the post-intervention groups for patients with preoperative pain above a cutoff of NRS_rest_>3 or NRS_movement_>5 (“patients with significant preoperative pain”) and for patients with pain below cutoff (NRS_rest_≤3 / NRS_movement_≤5; (“patients without significant preoperative pain”) before surgery for the total sample and for selected surgical groups (Table 4).

**Table 4:** Pain at rest and pain during movement in the pre-intervention and post-intervention phase in patients with preoperative pain below cutoff and patients with preoperative pain above cutoff.

Differences in Pain Intensity (NRS^a^)	Pain at rest			Pain during movement		
	Pre-test mean rank	Post-test mean rank		Pre-test mean rank	Post-test mean rank	
	(x_0.5_, n_valid_)	(x_0.5_, n_valid_)	Z, p	(x_0.5_, n_valid_)	(x_0.5_, n_valid_)	Z, p
**Total Sample**						
Pre ≤ cutoff	640.0 (1.5, n = 510)	583.9 (1.0, n = 704)	-2.835, p≈0.005▼ **	611.3 (3.0, n = 505)	561.4 (3.0, n = 660)	-2.531, p≈0.011▼ *
Pre > cutoff	194.2 (3.0, n = 182)	193.8 (3.0, n = 205)	-0.034, p≈0.973	206.2 (5.0, n = 181)	212.9 (5.0, n = 238)	-0.564, p≈0.573
**Joint (l)**						
Pre ≤ cutoff	52.9 (2.0, n = 62)	42.1 (2.0, n = 35)	-1.854, p≈0.064▼ ^T^	38.2 (4.0, n = 50)	36.0 (3.5, n = 24)	-0.407, p≈0.684
Pre > cutoff	36.2 (3.0, n = 46)	48.3 (4.0, n = 36)	-2.297, p≈0.022▲ *	49.0 (5.0, n = 57)	53.6 (5.0, n = 44)	-0.788, p≈0.431
**Thorax (s, m, l)**						
Pre ≤ cutoff	41.3 (2.0, n = 27)	29.1 (1.0, n = 40)	-2.575, p≈0.010▼ *	35.5 (3.0, n = 27)	32.1 (3.0, n = 39)	-0.705, p≈0.481
Pre > cutoff	---	---	---	---	---	---
**Visceral (m)**						
Pre ≤ cutoff	40.3 (1.0, n = 49)	42.1 (2.0, n = 32)	-0.340, p≈0.716	46.6 (4.0, n = 53)	39.9 (3.5, n = 34)	-1.222, p≈0.222
Pre > cutoff	23.2 (2.5, n = 30)	21.1 (2.0, n = 14)	-0.500, p≈0.617	19.0 (4.5, n = 28)	23.9 (5.5, n = 12)	-1.236, p≈0.216
**Tumour (s)**						
Pre ≤ cutoff	92.1 (1.0, n = 76)	72.5 (0.0, n = 87)	-2.935, p≈0.003▼ **	90.4 (2.0, n = 75)	73.8 (1.0, n = 87)	-2.307, p≈0.021▼ *
Pre > cutoff	---	---	---	---	---	---

Notes:

^▼^ significant reduction,

~ no difference,

^▲^ significant increase

*** p<0.001,

** p<0.01,

* p<0.05,

^T^ p<0.10,

(s) small, (m) medium, (l) major,

^a^ Nonparametric Mann-Whitney-U-Test is used to test differences between ordinal distributions

In the total sample, 45.4% (n = 182/401) of patients in the pre-intervention group reported significant preoperative pain at rest before surgery whereas 44.3% (n = 205/463) of patients in the post-intervention group reported significant preoperative pain. Preoperative pain during movement above cutoff was reported by 46.6% (n = 185/397) in the pre-intervention and 52.8% (n = 243/460) in the post-intervention group. Patients without significant preoperative pain experienced significantly lower postoperative pain at rest as well as during movement in the post-intervention versus the pre-intervention group. In contrast, there was no difference in postoperative pain at rest and during movement in patients with significant preoperative pain in the pre- versus the post-intervention group (Table 4).

In patients with major joint surgery (Table 4), postoperative pain at rest was significantly lower in the post- compared to the pre-intervention group only in patients without significant preoperative pain. In fact, patients with significant preoperative pain had higher pain at rest in the pre- compared to the post-intervention group. After thoracic surgery, patients without preoperative pain had lower pain after the intervention. Postoperative pain in patients with preoperative pain was similar in the pre- and post-intervention groups.

After visceral surgeries, no significant differences in pain intensity—neither for postoperative pain at rest, nor for pain during movement—were observed regardless if patients had preoperative pain above cutoff, or not.

#### Pain Management associated Side Effects and Symptoms

Patients after thoracic procedures reported less severe nausea, vomiting and fatigue by trend (p<0.10) (Table 5). In patients with skin tumour surgeries, the severity of fatigue and dizziness was significantly reduced (p<0.01) the post- versus the pre-intervention group. Based on the total sample of selected subgroups, significant reductions were observed for fatigue (p<0.05) and shaking while walking (p<0.01) in the post versus the pre-intervention group (Table 5).

**Table 5:** Severity of symptoms associated with pain and pain treatment for surgical subgroups with differences in pain ratings after intervention.

	Differences in Severity of Affliction^a^			
Surgeries^b^	Pre-test mean rank	Post-test mean rank		
	(x_0.5_, n_valid_)	(x_0.5_, n_valid_)	Z, p	▼ ~ ▲
**Joint (s)**				
Nausea	46.4 (0.0, n = 26)	44.4 (0.0, n = 63)	-0.577, p≈0.564	~
Vomiting	44.5 (0.0, n = 26)	45.2 (0.0, n = 63)	-0.642, p≈0.521	~
Fatigue	40.1 (0.0, n = 25)	46.2 (0.0, n = 63)	-1.142, p≈0.253	~
Dizziness	40.8 (0.0, n = 26)	46.7 (0.0, n = 63)	-1.234, p≈0.217	~
Shakiness	41.8 (0.0, n = 24)	44.8 (0.0, n = 63)	-0.646, p≈0.518	~
**Thorax (s, m, l))**				
Nausea	39.6 (0.5, n = 28)	31.8 (0.0, n = 41)	-1.829, p≈0.067	▼ ^T^
Vomiting	38.7 (0.0, n = 27)	31.7 (0.0, n = 41)	-1.954, p≈0.051	▼ ^T^
Fatigue	40.0 (2.0, n = 28)	31.6 (1.0, n = 41)	-1.782, p≈0.075	▼ ^T^
Dizziness	38.4 (1.0, n = 28)	31.8 (0.0, n = 40)	-1.529, p≈0.126	~
Shakiness	38.5 (1.0, n = 28)	32.7 (0.0, n = 41)	-1.310, p≈0.190	~
**Tumour (skin) (s)**				
Nausea	86.1 (0.0, n = 77)	83.2 (0.0, n = 91)	-0.676, p≈0.499	~
Vomiting	84.9 (0.0, n = 78)	84.2 (0.0, n = 91)	-0.233, p≈0.816	~
Fatigue	94.9 (0.0, n = 78)	76.6 (0.0, n = 91)	-2.960, **p≈0.003**	▼ **
Dizziness	92.2 (0.0, n = 77)	79.0 (0.0, n = 92)	-2.835, **p≈0.005**	▼ **
Shakiness	90.5 (0.0, n = 78)	81.3 (0.0, n = 92)	-1.696, **p≈0.090**	▼ ^T^
**Total**				
Nausea	560.3 (0.0, n = 539)	544.1 (0.0, n = 564)	-1.103, p≈0.270	~
Vomiting	546.6 (0.0, n = 536)	554.3 (0.0, n = 564)	-0.694, p≈0.488	~
Fatigue	570.2 (1.0, n = 538)	531.6 (0.0, n = 562)	-2.152, **p≈0.031**	▼ *
Dizziness	558.5 (0.0, n = 538)	544.8 (0.0, n = 564)	-0.891, p≈0.373	~
Shakiness	562.2 (0.0, n = 518)	511.5 (0.0, n = 553)	-3.130, **p≈0.002**	▼ **

Notes:

^▼^ significant reduction,

^~^ no difference,

^▲^ significant increase,

*** p<0.001,

** p<0.01,

* p<0.05,

^T^ Trend p<0.10,

(s) small, (m) medium, (l) major,

^a^ Nonparametric Mann-Whitney-U-Test is used due to ordinal measures,

^b^ Only surgeries with significant differences in pain at rest or pain during movement are displayed.

## Discussion

This is the first multicentre study prospectively investigating the effect of a perioperative pain improvement approach in a pre-post design based on patient-related outcome. Our data show an improvement of pain ratings after the intervention in the total sample and in select subgroups of patients after certain surgical procedures, e.g. small surgical procedures. In addition we found a consistent improvement of pain ratings in patients without pre-existing chronic pain before surgery but not in patients with pre-existing chronic pain. Finally, potential side effects of analgesics were reduced in the post-test sample indicating that improvement in pain management might not be associated with more side effects.

### Improvement of Pain Intensity

Our study was successful in improving acute pain after surgery in patients from six hospitals with a wide and typical spectrum of different surgical procedures in a medium size city (approximate population 300,000) in Germany. We included patients regardless of the type or size of the surgical procedure. However, to acknowledge a possible imbalance of procedures, we performed subgroup analyses with regard to the type of the surgical procedure. Median pain scores were significantly lower in the post-test group compared to the pre-test group in patients after small joint surgery, thoracic surgery and skin tumour excision. Thus, direct comparison between groups of patients with comparable surgical procedures further indicates an improvement due to the intervention and supports the effect of the intervention. The improvements are of clinical relevance, for instance if the numbers of patients with pain ratings above cutoff values are compared (Table 3).

Breaking it down to single OPS-defined surgical procedures, we were able to compare some different surgical subgroups with sufficient numbers of patients. Again, pre-post comparison of single procedures showed a meaningful decrease in pain ratings in the post-intervention skin cancer subgroup, a small but insignificant decrease in the thyroidectomy. Whereas the first two examples indicate success of the intervention, the latter shows difficulties in improvement in certain surgical subgroups (e.g. gynaecological surgery [13]). However, it supports the suggestion that the increase in pain ratings in patients with gynaecological procedures in the post-intervention group might result from an imbalance of gynaecological surgical procedures in the pre and post-intervention group. We were able to show that pain intensity scores after more minor surgical procedures improved, but most major surgical procedures (e.g. major orthopaedic or major visceral surgical procedures) remained unchanged mainly due low pain scores already in the pre-test sample (median pain intensity score of 2 at rest and 4 during movement).

Although significant, the reduction in pain ratings was in some surgical groups rather small. One plausible explanation are the low median pain scores before intervention in our pre-test sample (NRS = 2 at rest and NRS = 4 during movement) indicating an already successful pain management for these procedures before intervention. In fact, these pain scores are comparable to those in single centre studies after an intervention (mean of 1.6 at rest and 3.6 during movement) [19] and much lower compared to pain ratings before intervention [19] or in studies evaluating the “status quo” of pain outcome variables after surgery without intervention [8, 13]. For example, Gerbershagen et al. [13] recently reported a median (maximum) pain score above 5.5 on an 11-point scale for many orthopaedic surgical and some gynaecological procedures. Similar, pain scores after orthopaedic and gynaecological procedures were highest—compared to other procedures—in our study. However, they were lower compared to Gerbershagen et al. [13] and others presumably due to a general improvement in pain management procedures [23, 24].

### Improvement of Pain Intensity in Patients with versus without Preoperative Pain

As indicated in a recent large data analysis, preoperative pain is one major risk factor for high pain scores after surgery irrespective of the type of the procedure [22]. Therefore, another subgroup analysis was performed here to investigate if patients with and without preoperative pain benefitted in the same way from the intervention. Similar to Gerbershagen [22] we were able to show that pain scores were higher in patients with compared to those without preoperative chronic pain. This supports the fact that patients with preoperative pain have a higher risk for intense pain after surgery [22]. What is new, however, is the fact that pain intensity scores in patients without preoperative pain but not in those with preoperative pain improved after our intervention in most surgical groups. Thus, our data indicate for the first time in a real life situation that patients with preoperative pain may also respond less well to standardised pain management strategies (intervention) compared to those without preoperative pain. Furthermore, this might explain why pain ratings in patients after hip surgery and other major joint surgeries do not improve in the post-test after intervention: many patients receiving major joint surgery have chronic pain before surgery and pain is one of the major indications for surgery. The question arises, why patients with preoperative pain are not responding well to pain management improvement programmes. We actually addressed some of the aspects related to chronic pain patients within our interventional teaching programme. For example, we mentioned the phenomena “preoperative pain” and “preoperative opioids” as a ‘yellow flag’ for difficult pain management and addressed the question how to use regular pain medication in these patients in the perioperative period [25–28]. However, our data indicate that an improvement in patients with preoperative pain still proves difficult. Additional strategies for patients with chronic preoperative pain are needed. Perioperative pharmacological strategies and regional analgesia techniques may be incorporated more stringently as suggested by some pharmacological studies [26, 29–31] or systematic reviews [32]. Furthermore, the high impact of psychosocial aspects in patients with preoperative chronic pain [17] underlines the need for psychological approaches in the perioperative period for some patients with preoperative pain. Together, our data indicate that under real-life conditions, chronic preoperative pain is not only a simple risk factor for high pain ratings after surgery, but is an additional essential determinant for success of a quality improvement strategy. More specific and patient-oriented strategies are needed for patients with such special needs.

### Side Effects of Pain Medication

A successful pain improvement project should imply that side effects are not increased. Reducing pain scores may increase symptoms which are most likely related to analgesics’ side effects because more drugs are being applied. Although we were able to improve pain scores, typical side effects of analgesia were either lower or statistically indifferent in the post-intervention group. Interestingly, in patients after thoracic procedures (where pain ratings were reduced in the post-intervention group) nausea, vomiting and fatigue were lower in the post-intervention group indicating that pain reduction was not accompanied by more side effects. The most likely explanation is that strong opioids were administered related to the patients’ individual need after intervention. Furthermore, oral opioids were administered more often and intravenous opioids were administered less frequently after intervention. Thus, higher opioid doses inducing side effects were avoided explaining reduced incidence and severity of side effects. Pain management which is basically adapted to the patients’ needs is not and should not be automatically associated with an increase in side effects.

### Limitations and Future Directions

The strength of our study is the pre-post-design under real-life conditions rarely applied to examine in a multicentre approach an improvement in pain management after surgery. However, one limitation of this design is that we evaluated a non-random sample to channel a representative real-life sample of patients. The non-random sampling caused heterogeneity of procedures limiting comparison of the total group of patients. However, we tried to overcome this limitation by comparing results from certain types of procedures. Furthermore, it was only possible to analyse some specified sub-groups of patients after certain surgical procedures due to small sample sizes.

Nonetheless, due to the non-random selection procedure we cannot rule out the possibility of certain confounders. Because sample characteristics differed significantly between pre- and post-test, we’ve applied further multivariate statistics to address this issue. Only the significant reductions of pain intensity in patients with skin tumour surgical procedures seem to be confounded by sample characteristics—fractional by age and sex, but especially by the differing prescription of PRN analgesics before and after intervention. In fact, we interpret this as a positive effect with regards to content: We have reason to believe that this is to a significant part because of our introduced intervention and training programme, whereas the necessity of a target-oriented PRN usage against the background of the individuals’ pain situation was specifically highlighted. However, further potential confounders (e.g. attitudes toward pain and pain medication, interactions of analgesics, pain biographies) were neither controlled nor assessed and should be controlled in future studies.

Similar to most other studies, we examined postoperative pain only on the first day after surgery and only in hospitalised patients. These aspects should be focused on in the future. The described intervention was adapted to the results and circumstances in the individual hospital. In fact, after analysing the pre-test results and protocols, we had to deal with different problems in pain management in each particular hospital. Regional analgesia techniques were included in all hospitals from the beginning, e.g. for major orthopaedic and visceral procedures. However, aspects related to the management of pain after more minor or medium size procedures differed significantly. Therefore, aspects related to optimal measures of pain, cutoff points for intervention and an algorithm for treating pain after surgical procedures without regional analgesia techniques was main part of the intervention and trained individually. Inasmuch, the intervention was not explicitly standardised but very specific to the individual situation at hand in each hospital. Further studies are now needed to properly characterise important aspects relevant to a successful intervention programme.

## Supporting Information

S1 File Minimum Data Set “MDS_PogatzkiZahn_etal.sav” (Source: SPSS 21.0). (SAV)

S1 Table Characteristics of participants for total sample. (DOCX)

S2 Table Characteristics of participants for 12 surgery groups. (DOCX)

S3 Table Postoperative pain ratings (NRS) at rest and pain during movement in the pre-intervention and post-intervention group–single procedures. (DOCX)
