# Supplementary material for: A Prospective Multicentre Study to Improve Postoperative Pain: Identification of Potentialities and Problems
Source: PLoS One. 2015 Nov 24;10(11):e0143508. doi: 10.1371/journal.pone.0143508 (PMC4658204; doi:10.1371/journal.pone.0143508)
Supplement: S1 Table — (DOCX) [file pone.0143508.s002.docx]

S1 Table. Characteristics of participants for total sample

| **Characteristics of participants (n_valid_)** | | | |
| --- | --- | --- | --- |
| **Total Sample** | **Pre-test (n=708)** | **Post-test (n=935)** | **Pre- vs. Post-test^a^** |
| **Age** (n_pre_=706, n_post_=933), Mean (SD) | 53.60 (16.7) | 57.31 (17.5) | **p<0.001***** (t=-4.355) |
| 18-29 years | 11.3% (80) | 9.0% (84) |  |
| 30-59 years | 49.2% (347) | 42.0% (392) |  |
| 60-74 years | 30.6% (216) | 31.3% (292) |  |
| 75-95 years | 8.9% (63) | 17.7% (165) |  |
| **Sex** (n_pre_=708, n_post_=935) |  |  | **p≈0.034*** (chi²=4.476) |
| Male | 49.9% (353) | 44.6% (417) |  |
| Female | 50.1% (355) | 55.4% (518) |  |
| **BMI** (n_pre_=700, n_post_=908), Mean (SD) | 26.58 (5.0) | 26.62 (5.2) | n.s. |
| Underweight (<18.50) | 1.9% (13) | 1.7% (15) |  |
| Normal range (18.50-24.99) | 40.4% (283) | 40.9% (371) |  |
| Pre-obese (25.00-29.99) | 38.3% (268) | 38.0% (345) |  |
| Obese (≥ 30.00) | 19.4% (136) | 19.5% (177) |  |
| **Surgical Procedures** (n_pre_=704, n_post_=922, four most frequent) |  |  | **p<0.001***** (chi²=75.432) |
| Joint surgery | 23.2% (163) | 20.6% (190) |  |
| Visceral surgery | 14.5% (102) | 13.5% (124) |  |
| Tumor (skin) surgery | 11.1% (78) | 10.9% (100) |  |
| Gynaecological surgery | 9.1% (64) | 11.5% (106) |  |
| **Malignant Tumour** (n_pre_=707, n_post_=935) |  |  | **p≈0.043*** (chi²=4.076) |
| No | 76.8% (543) | 72.4% (677) |  |
| Yes | 23.2% (164) | 27.6% (258) |  |
| **Scheduled** **Analgesics** **–** **WHO** **level** (n_pre_=678, n_post_=931) |  |  | **p<0.001***** (chi²=103.937) |
| No analgesics | 8.0% (54) | 20.8% (194) |  |
| WHO level I | 45.6% (309) | 54.6% (508) |  |
| WHO level II & I+II | 14.0% (95) | 7.2% (67) |  |
| WHO level III & I+III | 32.4% (220) | 17.4% (162) |  |
| **PRN Analgesics – WHO level** (n_pre_=708, n_post_=935) |  |  | **p<0.001***** (chi²=57.548) |
| No analgesics | 45.7% (320) | 63.1% (589) |  |
| WHO level I | 20.1% (141) | 12.5% (117) |  |
| WHO level II & I+II | 7.7% (54) | 3.1% (29) |  |
| WHO level III & I+III | 26.4% (185) | 21.3% (199) |  |
| **Preoperative Pain at rest** (n_pre_=401, n_post_=463), Median | 3.0 | 3.0 | n.s. |
| > cutoff (NRS 3) | 45.4% (182) | 44.3% (205) |  |
| **Preoperative Pain during movement** (n_pre_=397, n_post_=460), Median | 5.0 | 6.0 | n.s. |
| >cutoff (NRS 5) | 46.6% (185) | 52.8% (243) |  |

Notes: *** p<0.001, ** p<0.01, * p<0.05, n.s. not significant, BMI Body Mass Index, PRN analgesics on demand, WHO World Health Organization, ^a^ t - Student t-test for independent variables, chi² - Pearson chi-square test
