# Supplementary material for: A Prospective Multicentre Study to Improve Postoperative Pain: Identification of Potentialities and Problems
Source: PLoS One. 2015 Nov 24;10(11):e0143508. doi: 10.1371/journal.pone.0143508 (PMC4658204; doi:10.1371/journal.pone.0143508)
Supplement: S2 Table — (DOCX) [file pone.0143508.s003.docx]

S2 Table. Characteristics of participants for 12 surgery groups

| **Characteristics of selected samples – Descriptive statistics for pre- and post-test** | | | | | | | | |
| --- | --- | --- | --- | --- | --- | --- | --- | --- |
| **Surgeries** | **Age (Mean, SD)** | | **Sex (% Female)** | | **Scheduled Analgesics (WHO level)** | | **PRN Analgesics (WHO level)** | |
|  | Pre | Post | Pre | Post | Pre | Post | Pre | Post |
| **Joint (s)** | 50.58 | 53.98 | 42.3% | 35.9% | No: 4.2% | No: 1.6% | No: 52.0% | No: 60.9% |
| (n=26/64) | (16.6) | (16.6) |  |  | I: 58.3% | I: 54.7% | I: 28.0% | I: 3.1% |
|  |  |  |  |  | II&I+II: 8.3% | II&I+II: 18.8% | II&I+II: 4.0% | II&I+II: 3.1% |
|  |  |  |  |  | III&I+III: 29.2% | III&I+III: 25.0% | III&I+III: 16.0% | III&I+III:32.8% |
| **Joint (m)** | 50.07 | 49.76 | 42.9% | 42.6% | No: --- | No: 3.8% | No: 66.7% | No: 61.1% |
| (n=28/54) | (18.4) | (16.2) |  |  | I: 48.1% | I: 64.2% | I: 14.8% | I: 3.7% |
|  |  |  |  |  | II&I+II: 18.5% | II&I+II: 11.3% | II&I+II: --- | II&I+II: 1.9% |
|  |  |  |  |  | III&I+III: 33.3% | III&I+III: 20.8% | III&I+III: 18.5% | III&I+III: 33.3% |
| **Joint (l)** | 58.43 | 68.28 | 45.9% | 70.8% | No: 2.2% | No: --- | No: 44.3% | No:54.9% |
| (n=109/71) | (16.9) | (12.9) |  |  | I: 26.1% | I: 25.7% | I: 11.3% | I: 2.8% |
|  |  |  |  |  | II&I+II: 21.7% | II&I+II: 42.9% | II&I+II: 0.9% | II&I+II: 4.2% |
|  |  |  |  |  | III&I+III: 50.0% | III&I+III: 31.4% | III&I+III: 43.4% | III&I+III: 38.0% |
| **Long bone (m, l)** | 53.92 | 61.90 | 43.6% | 76.2% | No: 2.7% | No: 4.8% | No: 40.5% | No: 47.6% |
| (n=39/21) | (19.6) | (16.5) |  |  | I: 43.2% | I: 38.1% | I: 21.6% | I: 9.5% |
|  |  |  |  |  | II&I+II: 2.7% | II&I+II: 9.5% | II&I+II: --- | II&I+II: --- |
|  |  |  |  |  | III&I+III: 51.4% | III&I+III: 47.6% | III&I+III: 37.8% | III&I+III: 42.9% |
| **Spine (m)** | 48.82 | 58.77 | 28.6% | 34.6% | No: --- | No: 7.7% | No: 48.1% | No: 42.3% |
| (n=28/26) | (11.0) | (13.0) |  |  | I: 41.7% | I: 53.8% | I: 22.2% | I: 3.8% |
|  |  |  |  |  | II&I+II: 29.2% | II&I+II: 15.4% | II&I+II: 3.7% | II&I+II: --- |
|  |  |  |  |  | III&I+III: 29.2% | III&I+III: 23.1% | III&I+III: 25.9% | III&I+III: 53.8% |
| **Thorax (s, m, l)** | 48.79 | 53.70 | 71.4% | 74.4% | No: 3.6% | No: -- | No: 32.1% | No: 79.1% |
| (n=28/43) | (16.8) | (14.7) |  |  | I: 42.9% | I: 95.3% | I: 21.4% | I: 11.6% |
|  |  |  |  |  | II&I+II: --- | II&I+II: --- | II&I+II: --- | II&I+II: --- |
|  |  |  |  |  | III&I+III: 53.6% | III&I+III: 4.7% | III&I+III: 46.4% | III&I+III: 9.3% |
| **Vascular (s, m)** | 71.00 | 61.29 | 20.0% | 42.9% | No: 14.3% | No: 61.9% | No: 40.0% | No: 71.4% |
| (n=15/21) | (4.7) | (15.5) |  |  | I: 50.0% | I: 38.1% | I: 40.0% | I: 14.3% |
|  |  |  |  |  | II&I+II: 7.1% | II&I+II: --- | II&I+II: 6.7% | II&I+II: 4.8% |
|  |  |  |  |  | III&I+III: 28.6% | III&I+III: --- | III&I+III: 13.3% | III&I+III: 9.5% |
| **Visceral (m)** | 49.63 | 53.51 | 44.6% | 72.3% | No: 6.0% | No: 6.4% | No: 39.8% | No: 68.1% |
| (n=82/47) | (14.2) | (17.3) |  |  | I: 50.6% | I: 68.1% | I: 22.9% | I: 12.8% |
|  |  |  |  |  | II&I+II: 1.2% | II&I+II: --- | II&I+II: 1.2% | II&I+II: --- |
|  |  |  |  |  | III&I+III: 42.2% | III&I+III: 25.5% | III&I+III: 36.1% | III&I+III: 19.1% |
| **Gynaecology (m)** | 47.98 | 45.06 | 100.0% | 100.0% | No: 11.1% | No: 13.7% | No: 40.7% | No: 52.9% |
| (n=54/51) | (14.1) | (14.2) |  |  | I: 66.7% | I: 64.7% | I: 40.7% | I: 23.5% |
|  |  |  |  |  | II&I+II: --- | II&I+II: --- | II&I+II: --- | II&I+II: --- |
|  |  |  |  |  | III&I+III: 22.2% | III&I+III: 21.6% | III&I+III: 18.5% | III&I+III: 23.5% |
| **Urology (m, l)** | 64.31 | 69.84 | 16.3% | 21.1% | No: 34.9% | No: 50.0% | No:72.1% | No: 63.2% |
| (n=42/38) | (12.8) | (10.8) |  |  | I: 37.2% | I: 26.3% | I: 4.7% | I: 23.7% |
|  |  |  |  |  | II&I+II: --- | II&I+II: --- | II&I+II: --- | II&I+II: --- |
|  |  |  |  |  | III&I+III: 27.9% | III&I+III: 23.7% | III&I+III: 23.3% | III&I+III: 13.2% |
| **Plastic (skin) (s)** | 48.64 | 52.30 | 35.7% | 62.5% | No: --- | No: 22.5% | No: 21.4% | No: 85.0% |
| (n=14/40) | (16.9) | (17.5) |  |  | I: 21.4% | I: 70.0% | I: 21.4% | I: 12.5% |
|  |  |  |  |  | II&I+II: 71.4% | II&I+II: 2.5% | II&I+II: 57.1% | II&I+II: --- |
|  |  |  |  |  | III&I+III: 7.1% | III&I+III: 5.0% | III&I+III: --- | III&I+III: 2.5% |
| **Tumour (skin) (s)** | 57.14 | 67.91 | 50.0% | 41.0% | No: 11.5% | No: 62.0% | No: 48.7% | No: 75.0% |
| (n=78/100) | (14.6) | (13.6) |  |  | I: 55.1% | I: 35.0% | I: 19.2% | I: 15.0% |
|  |  |  |  |  | II&I+II: 33.3% | II&I+II: --- | II&I+II: 32.1% | II&I+II: 9.0% |
|  |  |  |  |  | III&I+III: --- | III&I+III: 3.0% | III&I+III: --- | III&I+III: 1.0% |
| **Total (surgeries)** | 54.32 | 58.62 | 48.3% | 55.8% | No: 8.1% | No: 20.7% | No: 46.2% | No: 64.8% |
| (n=543/576) | (16.3) | (16.9) |  |  | I: 45.6% | I: 51.6% | I: 20.5% | I: 11.1% |
|  |  |  |  |  | II&I+II: 14.1% | II&I+II: 9.6% | II&I+II: 7.1% | II&I+II: 2.8% |
|  |  |  |  |  | III&I+III: 32.2% | III&I+III: 18.1% | III&I+III: 26.3% | III&I+III: 21.4% |

Notes: (s) small, (m) medium, (l) major surgery, PRN analgesics on demand
