# Supplementary material for: A Prospective Multicentre Study to Improve Postoperative Pain: Identification of Potentialities and Problems
Source: PLoS One. 2015 Nov 24;10(11):e0143508. doi: 10.1371/journal.pone.0143508 (PMC4658204; doi:10.1371/journal.pone.0143508)
Supplement: S3 Table — (DOCX) [file pone.0143508.s004.docx]

S3 Table. Postoperative pain ratings (NRS) at rest and pain during movement in the pre-intervention and post-intervention group – single procedures

| **Surgeries** | **Differences in pain intensity – NRS^a^** | | | | **NRS-cutoff^b^** | | |
| --- | --- | --- | --- | --- | --- | --- | --- |
|  | **Pre-test** | **Post-test** |  |  |  |  |  |
|  | **mean rank** | **mean rank** |  |  | **% > cutoff** |  |  |
|  | **(x_0.5_, n_valid_)** | **(x_0.5_, n_valid_)** | **Z, p** | ***▼~▲*** | **Pre vs. Post** | **V** | **p (chi²)** |
| **Hip joint replacement** |  |  |  |  |  |  |  |
| Pain at rest | 24.4 (1.0, n=23) | 34.9 (3.0, n=38) | -2.278, p≈0.023 | ***▲**** | 17.4 vs. 34.2% | 0.182 | p≈0.156 (2.02) |
| Pain during movement | 25.8 (4.0, n=22) | 32.5 (5.0, n=37) | -1.443, p≈0.149 | ~ | 31.8 vs. 45.9% | 0.139 | p≈0.285 (1.14) |
| **Thyroid surgery** |  |  |  |  |  |  |  |
| Pain at rest | 28.8 (2.0, n=22) | 21.9 (1.0, n=27) | -1.713, p≈0.087 | **▼^T^** | 18.2 vs. 14.8% | 0.045 | p≈0.751 (0.10) |
| Pain during movement | 24.3 (3.5, n=22) | 25.6 (3.0, n=27) | -0.325, p≈0.745 | ~ | 18.2 vs. 22.2% | 0.050 | p≈0.727 (0.12) |
| **Hysterectomy** |  |  |  |  |  |  |  |
| Pain at rest | 31.2 (3.0, n=26) | 28.2 (2.0, n=32) | -0.682, p≈0.495 | ~ | 30.8 vs. 31.2% | 0.005 | p≈0.969 (0.01) |
| Pain during movement | 27.2 (4.5, n=82) | 30.6 (5.0, n=31) | -0.775, p≈0.438 | ~ | 34.6 vs. 35.5% | 0.009 | p≈0.945 (0.01) |
| **Excisions from skin and hypoderm** |  |  |  |  |  |  |  |
| Pain at rest | 91.3 (1.0, n=58) | 69.2 (0.0, n=96) | -3.176, **p<0.001** | **▼***** | 19.0 vs. 6.2% | **0.197*** | **p≈0.015** (5.95) |
| Pain during movement | 86.6 (2.0, n=56) | 69.8 (1.0, n=95) | -2.335, **p≈0.020** | **▼*** | 14.3 vs. 9.5% | 0.074 | p≈0.366 (0.82) |

Notes: ▼ significant reduction, ~ no difference, ▲ significant increase, *** p<0.001, ** p<0.01, * p<0.05, ^T^ p<0.10
^a^ Nonparametric Mann-Whitney-U-Test is used to test differences between ordinal distributions, ^b^ Pearson chi-square and Cramer V are used to test differences between dichotomous distributions (pain at rest>3, pain during movement>5)
